# Impact of contact data resolution on the evaluation of interventions in mathematical models of infectious diseases

**DOI:** 10.1098/rsif.2022.0164

**Published:** 2022-06-22

**Authors:** Diego Andrés Contreras, Elisabetta Colosi, Giulia Bassignana, Vittoria Colizza, Alain Barrat

**Affiliations:** ^1^ Aix Marseille University, Université de Toulon, CNRS, CPT, Turing Center for Living Systems, Marseille, France; ^2^ INSERM, Sorbonne Université, Pierre Louis Institute of Epidemiology and Public Health, Paris, France; ^3^ Tokyo Tech World Research Hub Initiative (WRHI), Tokyo Institute of Technology, Tokyo, Japan

**Keywords:** epidemiology, COVID-19, data-driven modelling, complex networks

## Abstract

Computational models offer a unique setting to test strategies to mitigate the spread of infectious diseases, providing useful insights to applied public health. To be actionable, models need to be informed by data, which can be available at different levels of detail. While high-resolution data describing contacts between individuals are increasingly available, data gathering remains challenging, especially during a health emergency. Many models thus use synthetic data or coarse information to evaluate intervention protocols. Here, we evaluate how the representation of contact data might affect the impact of various strategies in models, in the realm of COVID-19 transmission in educational and work contexts. Starting from high-resolution contact data, we use detailed to coarse data representations to inform a model of SARS-CoV-2 transmission and simulate different mitigation strategies. We find that coarse data representations estimate a lower risk of superspreading events. However, the rankings of protocols according to their efficiency or cost remain coherent across representations, ensuring the consistency of model findings to inform public health advice. Caution should be taken, however, on the quantitative estimations of those benefits and costs triggering the adoption of protocols, as these may depend on data representation.

## 1. Introduction

Computational models and numerical simulations are essential tools for the understanding of epidemic spread [1,2], at scales ranging from global to local [3–6]. They have been used in the past to examine pandemic scenarios, and more extensively during the current COVID-19 pandemic, to evaluate the potential impact of non-pharmaceutical interventions (NPIs) ranging from international travel restrictions [4,5,7–9] to lockdowns or curfews aiming at reducing global mobility and interactions [10–13], to more targeted measures such as isolation of positive cases, contact tracing, telework, partial closures of schools or surveillance by regular testing [14–22].

Epidemic models of infectious diseases rely both on the disease progression within hosts and on the description of how the disease can propagate from host to host, i.e. of the interactions between hosts. These interactions can be described at various levels of detail: at the coarsest level, homogeneous mixing [1] assumes that individuals potentially interact with others in a uniform way; contact matrices divide individuals into classes, and give the average duration of contacts between individuals of given classes [23]; contact networks describe specifically which pairs of hosts are in contact [24–26]. Regardless of the level of description chosen, a model needs to be informed by data in order to be actionable, i.e. to provide scenarios that can inform public health decisions. These data are typically collected by surveys or diaries [23,27–29] or, more recently, using wearable sensors able to detect close-range proximity between individuals with high spatial and temporal resolution [30–34].

Gathering data is, however, expensive, time-consuming and implies logistical challenges, which become particularly prohibitive for large-scale populations or multiple coupled settings, especially for high-resolution data [25,35]. The question of how much detail should be included in computational models arises, therefore, naturally [6,28,36]. For instance, the estimation of superspreading events needs to be informed by the heterogeneity of contact patterns [37]. Coarse representations can also yield higher estimates of epidemic risk and attack rates of specific groups than more detailed representations [6,38,39], even if a rescaling of parameters can enhance the accuracy of models based on a homogeneous mixing hypothesis [40]. To overcome the limitations of coarse representations, intermediate data representations informed by statistical heterogeneities of contact numbers and durations, and yielding a good estimation of the epidemic risk, have been put forward [38,39].

Although data with a limited resolution were shown to be insufficient to inform interventions at individual scale [41], they are still useful to inform strategies at intermediate scales [14,15,42–44]. In practice, however, a general issue faced by models concerns the comparison of strategies or control measures, in terms of both costs and benefits. In the case of COVID-19 for instance, the computational models mentioned above have considered a wide variety of measures (contact tracing, regular testing, telework, class or school closures), with each study using specific empirical or synthetic data and a specific representation of contacts [17,19–22,44–49]. However, just as the data representation can affect the identification of risk groups [38], it might also impact the assessment of different strategies. Here we tackle this issue by leveraging high-resolution data describing contacts between individuals in several settings (offices, schools, hospital). We consider several representations of the data, from fine-detailed to coarse-grained ones [38], and use them to inform an agent-based model of SARS-CoV-2 transmission in these settings. We simulate several strategies (reactive and regular testing, telework, reactive class closures) and evaluate their cost and benefit for each representation, highlighting differences and similarities in the outcomes.

## 2. Methods

We consider a model for SARS-CoV-2 spread in different settings, namely two schools, an office setting and a hospital ward. In this section, we first present the compartmental model used and the pharmaceutical (vaccination) and NPI considered. We then describe the high-resolution data on interactions between individuals that we use, as well as the hierarchy of coarse-grained representations of the contact patterns that preserve the temporal and structural information of the data at different levels of detail.

### 2.1. Compartmental model

**Figure 1.**
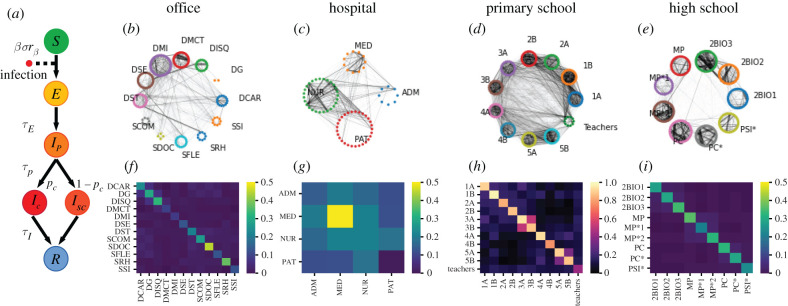
Model and datasets. (*a*) Schematic illustration of the epidemic model. After contact with an infectious individual, a susceptible individual can become exposed, then transition to a pre-symptomatic state. The individual can then develop either a clinical or a sub-clinical infection before recovering. (*b*–*e*) Weighted networks of contacts for the office, hospital, primary school and high school, respectively. For each setting, interactions are aggregated over the first data collection day. The width of an edge is proportional to its weight, i.e. the total contact time between the individuals connected. For each setting, the individuals belonging to the same category are represented in a circle; the categories correspond to: departments in the office, roles in the hospital (doctors, nurses, administrative staff and patients), classes in the school settings. (*f* –*i*) Contact matrices showing the average daily density of links between categories, respectively in the office, hospital, primary school and high school.

We use an agent-based model in which the progression of the disease within each host follows discrete states, as sketched in figure 1*a* [20]. Infectious individuals can transmit the disease to susceptible (healthy) individuals (*S*), who first enter the exposed (non-infectious) state (*E*) and then a pre-symptomatic infectious state (*I*_*p*_) after a time *τ*_*E*_. The pre-symptomatic phase lasts *τ*_*p*_, after which individuals either evolve into a sub-clinical infection (*I*_sc_) or manifest a clinical infection *I*_*c*_, with respective probabilities 1 − *p*_*c*_ and *p*_*c*_. The infectious state leads finally to the recovered state *R* after a time *τ*_*I*_. The disease state durations *τ*_*E*_, *τ*_*p*_ and *τ*_*I*_ are distributed according to Gamma distributions, with average values and standard deviations given in table 1 (see also electronic supplementary material, S1.2.4). We explore in electronic supplementary material, S2.5.1, a wide range of values of the infectious period *τ*_*p*_ + *τ*_*I*_ as sensitivity analysis.

**Table 1.** Parameters of the compartmental model, taken from [20].

SEIR parameter	value
	mean (s.d.) (days)
*τ*_*E*_	4 (2.3)
*τ*_*p*_	1.8 (1.8)
*τ*_*I*_	5 (2.0)
*R*_0_	1.5, 3.0
*p*_*c*_	0.5
*σ*	1.0
$r_{\beta }^{\;p}$, $r_{\beta }^{\mathrm{sc}}$	0.55
$r_{\beta }^{c}$	1.0

Transmission of the disease can occur upon contact between a susceptible and an infectious (*I*_*p*_, *I*_sc_ or *I*_*c*_). The probability of transmission per unit of time depends on the product of the transmission rate *β*, the relative infectiousness $r_{\beta }$ of the infectious individual and the susceptibility *σ* of the agent. The parameter *β* is tuned to obtain a desired specific value for the basic reproductive number *R*_0_, as detailed in electronic supplementary material, S1.3. The relative infectiousness $r_{\beta }$ depends on the compartment of the infectious individual, with a larger $r_{\beta }^{c}$ value for infectious individuals in the clinical state *I*_*c*_, and lower values $r_{\beta }^{\;p}$ and $r_{\beta }^{sc}$ for *I*_*p*_ and *I*_sc_ (table 1). It also depends on the age class of the infectious, with adults and adolescents more infectious than children (table 2). The susceptibility *σ* also depends on the age of the susceptible individual, with adults more susceptible than other groups (adolescents and children have a susceptibility reduced by, respectively, 25% and 50% with respect to adults; see table 2). Finally, the probability of developing a clinical infection is also reduced by 60% for both adolescents and children.

**Table 2.** Reduction in susceptibility *σ*, probability of clinical infection *p*_*c*_ and relative infectiousness $r_{\beta }$ for children and adolescents, with respect to their values for adults. Taken from [20].

parameter	reduction for children (%)	reduction for adolescents (%)
*σ*	50	25
*p*_*c*_	60	60
$r_{\beta }$	27	0

We can further enrich the compartmental model of figure 1*a* by considering that individuals can be vaccinated. Here, we do not consider a dynamic vaccination rollout, and assume that vaccination coverage is fixed throughout the simulation. We also assume full vaccination of individuals. We assume vaccination to reduce $r_{\beta }$ by 50%, *σ* by 85% and *p*_*c*_ by 93% We consider (in electronic supplementary material, S2.4) levels of vaccination coverage of 25%, 50% and 75%. As sensitivity analysis, we also consider a less effective vaccine (see electronic supplementary material, S2.5.4).

### 2.2. Non-pharmaceutical interventions

We consider several interventions based on testing and isolation of cases, as well as the closure of classes in school settings, and telework in offices.

We use as baseline the protocol of *symptomatic testing and case isolation:* clinical cases have a probability *p*_*D*_ = 0.5 (*p*_*D*_ = 0.3 for children) to take a test and then isolate for Δ_*Q*_ = 7 days after receiving the result of the test. Tests are performed outside work/school hours. Symptomatic individuals remain isolated while they wait for their test results. This protocol is used as a reference protocol against which all other protocols are compared.

With symptomatic testing and case isolation always implemented, we consider the following additional NPIs:

— *Regular testing:* Non-vaccinated individuals are periodically tested. We explore weekly, semiweekly (twice per week) or biweekly (once every two weeks) testing with an adherence *α* (fraction of the population accepting to get tested). Positive cases remain in isolation for Δ_*Q*_ = 7 d. Tests are performed during work/school hours.
— *Telework:* Telework is implemented only in the office setting. We explore weekly, semiweekly (twice per week) or biweekly (once every two weeks) telework. For each individual, we fix at random the days of the week in which they work remotely and have no contact with the other office workers.
— *Class quarantine:* This protocol is implemented only in the school settings. When an individual is tested positive upon symptomatic testing, the whole class goes into isolation for Δ_*Q*_ = 7 d.
— *Reactive testing:* This protocol is implemented in the school settings and in the office setting. When an individual tests positive upon symptomatic testing, the non-vaccinated students of the same class (for schools) or the members of the same department (for offices) are tested after a time Δ_*r*1_ = 1 d, with an adherence *α*. A second test is performed after Δ_*r*2_ = 4 d. Positive cases are quarantined during Δ_*Q*_ = 7 d.

In the office setting, we additionally consider a protocol in which regular testing is combined with telework. Further details of the implementation can be found in electronic supplementary material, S1.2.

The efficacy of a protocol is quantified in terms of relative reduction of cases with respect to the symptomatic testing protocol at the end of 60 simulation days. The cost is measured as the average number of days spent in quarantine per individual after 60 d. In addition, we measure the number of tests performed after 60 d. Costs and benefits are also evaluated at additional points in time (after 30, 90 or 120 d); see electronic supplementary material, S2.5.5.

In all scenarios, we consider self-administered antigenic tests with turnaround time Δ_*w*_ = 15 min [20]. We assume the tests to have a 100% specificity, and a sensitivity *θ* which depends on the infectious compartment, with *θ*_*p*_ = 0.5, *θ*_*c*_ = 0.8 and *θ*_sc_ = 0.7 for the pre-symptomatic, clinical and sub-clinical compartments, respectively. As sensitivity analysis, we consider in the electronic supplementary material the case of PCR tests with higher sensitivity and longer turnaround time (see electronic supplementary material, S2.5.2).

### 2.3. Empirical contact data

We use high-resolution face-to-face empirical contacts data collected using wearable sensors in four different settings, two workplaces and two educational contexts: an office building, a hospital, a primary school and a high school. The datasets are publicly available at http://www.sociopatterns.org/datasets.

— The office dataset gathers the contacts among 214 individuals, measured in an office building in France during two weeks in 2015 [41]. Individuals are divided in 12 departments with different sizes.
— The hospital dataset describes the interaction among 42 healthcare workers (HCWs) and 29 patients in a hospital ward in Lyon, France, gathered during 3 days in 2010 [32]. HCWs are divided in three roles: nurses, doctors and administrative staff.
— The primary school dataset describes the contacts among 232 children and 10 teachers in a primary school in Lyon, France, during 2 days of school activity in 2009 [42]. The school is composed of 5 grades, each of them comprising 2 classes, for a total of 10 classes; there is a teacher for each class.
— The high school dataset describes the contacts among 324 students of ‘classes préparatoires’ in Marseille, France, during one week in 2013 [50]. These classes are located in high schools and are specific to the French schooling system: they gather students for 2-year studies at the end of the standard curriculum to prepare for entry exams at specific universities. Students are grouped in nine different classes, and classes are divided in three groups, each focusing on a specialization (mathematics and physics; physics, chemistry, engineering studies; biology).

Datasets are available as lists of contacts over time between anonymized individuals, with a classification by department (for the office setting), role (for the hospital) or class (for the school settings), and in terms of students/teachers (for the primary school). From the raw data, we built the corresponding temporal contact networks, composed of nodes representing individuals and links representing empirically measured proximity contacts occurring at a given time (see electronic supplementary material, S1.1.1).

Figure 1*b*–*e* displays for each setting a graph of the links aggregated over 1 day for each dataset (where the weight of a link between two individuals is given by the total contact time between them). The corresponding contact matrices representing the daily average density of interactions are shown in figure 1*f*–*i*. In school settings and in offices, contacts occur preferentially within groups [41,42,50].

### 2.4. Data representations

The empirical data describe contacts at high resolution, giving temporally resolved information on who has been in contact with whom. These data can be aggregated into representations at different levels of detail, i.e. retaining only selected features of the empirical temporal contact network while aggregating over the others.

The first type of representations, which we denote by *individual-based representations*, preserve the empirical structure of the contact network (who has met whom).

— *Dynamical network:* Contacts are aggregated into a different weighted graph for each successive time window of 15 min (the weight of a link between two nodes is given by the time in contact of the two corresponding individuals during this time window). This representation is the closest to the raw empirical data (that has a temporal resolution of 20 s), and will be considered as the reference against which the other representations will be compared.
— *Heterogeneous network:* Contacts measured during the whole data collection are aggregated into a single weighted network. The weight of a link (*i*, *j*) is given by the average daily contact time between *i* and *j*.
— In addition, we consider in electronic supplementary material, S2, the *daily heterogeneous network representation:* contacts are aggregated into a different weighted graph for each of the *d*_data_ days of data collection. The weight *w*_*ij*,*d*_ of a link (*i*, *j*) on day *d* is given by the total contact time registered between *i* and *j* during the corresponding day.

In a second type of representations, the *category-based representations*, we aggregate individuals into categories, corresponding to departments for the office data, to roles for the hospital data, and to classes in the school settings (and a category for teachers in the primary school data). Individuals belonging to a given category are considered as *a priori* equivalent. For each pair of categories *X* and *Y*, we summarize the interactions between individuals of these categories by the list of daily contact weights *D*_*XY*_ = {*w*_*ij*,*d*_|*i* ∈ *X*, *j* ∈ *Y*, *d* ∈ [1, *d*_data_]}. The average daily number of links between individuals of categories *X* and *Y* is *E*_*XY*_ = |*D*_*XY*_|/*d*_data_, and the quantity $W_{XY}=\sum_{i\in X,j\in Y,d}w_{ij,d}/d_{\mathrm{data}}$ gives the average daily total time in contact between individuals of categories *X* and *Y*. We define the three following data representations based on the concept of contact matrix [38]:

— *Contact matrix:* Each individual from category *X* is connected with all individuals of category *Y* with a weight equal to *w*_*XY*_ = *W*_*XY*_/(*N*_*X*_ *N*_*Y*_) (*N*_*X*_ is the number of individuals in category *X*; for *X* = *Y* we set *w*_*XX*_ = *W*_*XX*_/(*N*_*X*_ (*N*_*X*_ − 1)/2)). This representation only retains the average time spent in contact between members of given categories. For instance in the hospital data, *W*_NUR,ADM_ gives the total contact time between nurses and members of the administrative staff.
— *Contact matrix of distributions:* This representation preserves the information about the density of links between categories and the statistical heterogeneity of the daily contact durations between pairs of individuals. First, we create for each day a random graph assigning *E*_*XY*_ random links connecting individuals of categories *X* and *Y*. The weight of each link between individuals of categories *X* and *Y* is then drawn from a negative binomial distribution, obtained by fitting the empirical distribution *D*_*XY*_ through a maximum-likelihood procedure. In the hospital data for instance, for the contacts between nurses and administrative staff members, this representation retains the actual average daily number *E*_NUR,ADM_ of links between these categories, and it also uses the fitted distribution of all observed daily contact times between nurses and staff members.
— In addition, we consider in electronic supplementary material, S2, the *contact matrix of bimodal distributions:* similarly to the contact matrix of distributions, this representation retains the information about the density of links between categories, but it disregards the heterogeneity of link weights. We thus create for each day a graph with *E*_*XY*_ random links connecting individuals of categories *X* and *Y*. However, only the average of each distribution *D*_*XY*_ is retained: each link is assigned a weight ${\tilde{w}}_{XY}=W_{XY}/E_{XY}$. In the hospital data for instance, ${\tilde{w}}_{\mathrm{NUR},\mathrm{ADM}}$ gives the average contact time on a link between a nurse and a member of the staff.

We also consider for reference a very coarse representation informed only by the total daily contact time:

— *Fully connected:* Individuals are all connected with each other. The weight of each link is equal to the daily contact time averaged over the whole dataset $w=\sum_{XY}W_{XY}/$ (N(N−1)/2), where $N=\sum_{X}N_{X}$ is the total number of individuals.

Only the dynamical network representation retains information on the temporal evolution of contact activity during each day. However, we inform all other representations by the office or school hours and by the alternation of weekdays and weekends, as reported in table 3: no contacts occur outside of these hours. In particular, no contacts occur during the weekends in the office and school settings. During the nights, weekends (and on Wednesdays for the primary school), nodes are thus isolated in the simulations.

**Table 3.** Number of days *d*_data_ of the dataset, number of individuals *N*, initial hour (*t*_*i*_) and final hour (*t*_*f*_) of each day, and days of activity in each week (indicated with an X) for each setting.

setting	*d*_data_	*N*	*t*_*i*_	*t*_*f*_	M	T	W	T	F	S	S
office	10	214	8.00	20.00	X	X	X	X	X		
hospital	3	71	5.00	00.00	X	X	X	X	X	X	X
primary school	2	242	8.30	17.15	X	X		X	X		
high school	4	324	9.00	18.00	X	X	X	X	X		

### 2.5. Simulation setup

Simulations are initialized at a random time with one exposed individual chosen at random. Simulations then unfold stochastically (see electronic supplementary material, S1.2), with transmission events occurring, for each representation, along the contacts available in that representation of the data. To simulate the disease spreading on longer time scales than the available data (table 3), copies of the initial data are repeated over time. Periodic introductions are considered to model infections from the community. At regular intervals a susceptible individual in the considered setting is chosen at random and switched to the exposed compartment (see electronic supplementary material, S1.2.5). To simulate a limited adherence to testing, the individuals accepting to perform tests are randomly chosen at the beginning of each simulation. Finally, we also explore in electronic supplementary material, S2.2, the effect of initial immunity, simulated by the fact that a fraction of the population, randomly chosen at the start of each simulation, cannot be contaminated.

As discussed in [38,39], simulations using a given rate of transmission *β* performed on different data representations yield different outcomes: less detailed representations tend to yield a higher epidemic final size compared to the dynamical network representation [38], as they make more transmission paths available. Therefore, we fix a target basic reproductive number *R*_0_ in the absence of any control measures and starting with one random seed in an otherwise susceptible population, and calibrate for each representation the rate of transmission *β* needed to obtain the target *R*_0_ (see electronic supplementary material, S1.3).

We consider two types of simulations. On the one hand, we study the dynamics of the spreading process in the absence of interventions, starting from one random seed and with no introductions, and running simulations until no infectious individual is present in the population (§3.1). Results are averaged over 2000 simulations, except the distributions of the number of secondary infections for which we use 6000 simulations. On the other hand, to evaluate NPIs, we consider in §3.2 simulations of a spread starting from one initial seed, with in addition biweekly introductions of exposed individuals. We simulate the spread for 60 d and compute the final epidemic size as well as the number of days that individuals spent in quarantine and the number of tests performed. Each result corresponds to a median over 2000 simulations, with bootstrapped confidence intervals (see electronic supplementary material, S1.4).

## 3. Results

### 3.1. Unmitigated spread on different data representations

We present here the results concerning the unmitigated spread with *R*_0_ = 3 in the office dataset, and we show in electronic supplementary material, S2.2, the results for the other datasets and both *R*_0_ = 1.5 and *R*_0_ = 3.

**Figure 2.**
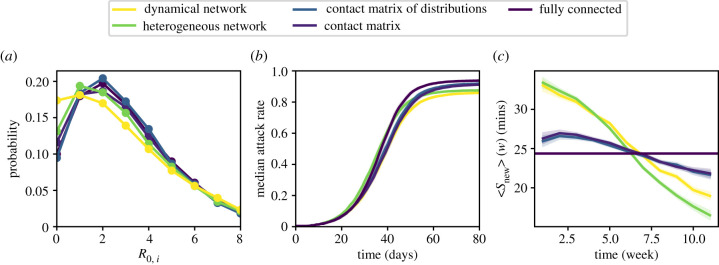
Spreading dynamics on different representations of the office dataset, for *R*_0_ = 3.0, starting from a single initial exposed seed and no initial immunity. (*a*) Distribution of the number of secondary infections produced by the initial seed. (*b*) Temporal evolution of the median attack rate (fraction of individuals who have been infected), starting from one single exposed individual in an otherwise susceptible initial population. (*c*) Average strength (daily time in contact) of newly infected individuals infected in a given week versus time. For individual-based representations, a cascade from more connected individuals to less connected ones is observed. The cascade is less pronounced for category-based representations and absent for the fully connected case. Shaded areas correspond to the estimated error, obtained as a bootstrapped CI (see electronic supplementary material, S1.4).

Figure 2 highlights differences and similarities between the processes taking place on different representations of the same dataset. Figure 2*a* shows the distributions of the number of secondary cases resulting from one random seed, *R*_0,*i*_ (the basic reproductive number *R*_0_, which takes by construction the same value in all cases, being the average of this distribution), obtained on the various data representations. All distributions span a rather wide range of values, with events reaching almost four times the average. However, the curves exhibit distinct shapes depending on the type of representation. In the category-based representations, both small and large values of *R*_0,*i*_ have a lower probability than for individual-based representations, i.e. both the probability that the spread never starts and the probability that superspreading events occur are lower. Fitting the distributions with negative binomials yields indeed values of the over-dispersion parameter *k* larger for the individual-based representations (≈0.5 for *R*_0_ = 3 in the office dataset; see electronic supplementary material, S2.2) than for the category-based ones (≈0.25 for the contact matrix representations and ≈0.22 for the fully connected representation, for *R*_0_ = 3 in the office dataset; see electronic supplementary material, table S4).

Another interesting difference between the two types of representations arises from the investigation of how the spread evolves within the population. Figure 2*b* shows the temporal behaviour of the fraction of infected individuals for the various representations. The growth is slightly faster at short times for individual-based representations with respect to category-based ones, saturating at earlier times and smaller final epidemic sizes. These differences in dynamics can be understood by examining which nodes are infected at early and late stages of the spread. Indeed, a spreading process on a network tends first to reach the most connected nodes, with a following cascade towards the less connected nodes, so that the average number of neighbours of newly infected nodes decreases with time [51]. Here, as heterogeneities concern contact times rather than numbers of neighbours [35], we show in figure 2*c* the average daily strength <*s*_new_> (*w*) of individuals who are infected and become exposed during week *w* (the strength *s* of an individual is the average daily time in contact with other individuals). The cascading process from individuals with large *s* towards individuals with lower *s* is seen as a decreasing trend of <*s*_new_> (*w*) for the individual-based representations. For the category-based representations, the cascade still exists, but the effect is weaker: all individuals within a category are equivalent, but some categories are more connected than others, so that some heterogeneity remains in the population. Overall, at early times the newly infected individuals are more connected in the individual-based representations than in category-based ones, leading to a faster spread. At later times, the tendency is inverted, with a slower spread on individual-based representations; moreover, as the remaining susceptible individuals tend to be less well connected, and as fewer paths are available to reach them, the final epidemic size is also smaller. On the other hand, simulations using the fully connected representation cannot show any such effect as all individuals are equivalent. An additional difference is observed between the heterogeneous network and the dynamical network representations: more causal propagation paths are present in the heterogeneous network case (where the same network of contacts is present every day) so that more nodes with smaller strength can be reached by the cascade and a larger epidemic size is obtained (as seen in figure 2*b*).

Similar results across representations are obtained considering a partially immune population (electronic supplementary material, S2.2).

### 3.2. Robustness of the evaluation of non-pharmaceutical interventions

We show here the results of simulations implementing NPIs for *R*_0_ = 1.5, and present additional results and sensitivity analysis in electronic supplementary material, S2.3–S2.5. We illustrate the numerical simulations in electronic supplementary material, videos SV1 and SV2: each video shows a single run in the office dataset, with the symptomatic testing protocol (SV1) and the regular testing protocol (SV2, with weekly testing and 75% adherence). In each video, we present side-by-side runs on three different representations of the data: the dynamical network, the heterogeneous network and the contact matrix of distributions. This shows how the links of the dynamical network change at every time step, while the heterogeneous network links are fixed (disappearing only during nights and weekends) and the links of the contact matrix of distributions representation are renewed daily.

**Figure 3.**
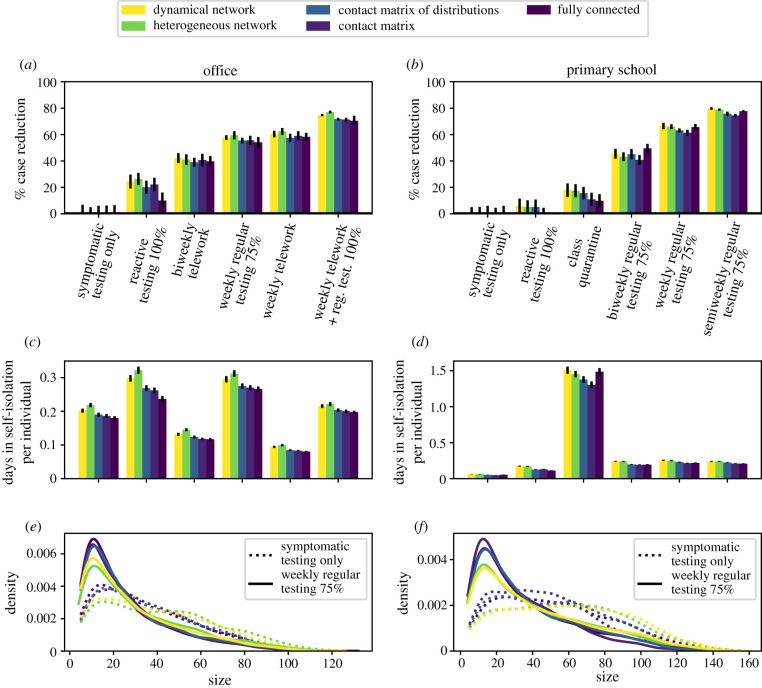
Evaluation of several NPIs in office and primary school settings, for *R*_0_ = 1.5 and simulations performed using different data representations. (*a*,*b*) Efficacy of NPIs in office and primary school, sorted by increasing order of efficacy in the dynamical network representation. Efficacy is defined as the relative reduction in median size compared with symptomatic testing alone, after a period of 60 d. (*c*,*d*) Average number of days in quarantine per individual under different protocols (same *x*-axis as *a* and *b*). (*e*,*f*) Epidemic size distributions for the symptomatic testing protocol (dotted lines), and for weekly regular testing with 75% adherence (continuous line).

We consider testing and isolation of symptomatic individuals to be the minimal strategy at play, and focus on a comparison of all protocols with respect to this strategy (the impact of this baseline intervention with respect to the absence of intervention is shown in electronic supplementary material, S2.3). We present the results for the office and primary school datasets in figure 3, and show the results for other datasets in electronic supplementary material, S2.3, as well as additional values of the protocols’ parameters. Figure 3*a*,*b* shows the reduction in the median epidemic size after 60 d for several protocols, with respect to the symptomatic testing, with protocols ranked in order of increasing reduction. Strikingly, even if the precise values of the efficacy of each protocol depend slightly on the data representation used in the simulations, the ranking of protocols remains almost always the same, for both benefits (figure 3*a*,*b*) and costs (figure 3*c*,*d*). In particular, telework in the office is particularly efficient, as it reduces the number of contacts of all individuals [19], whereas reactive strategies at school are less efficient than regular testing, because asymptomatic transmissions mostly go undetected, as shown in [20]. These conclusions are reached for all the data representations. Note that the robustness of the ranking with respect to the representation is very strong but not perfect: if two protocols yield very close average efficacy values, one can seem slightly better than the other for one representation and slightly worse for another. Moreover, some exceptions can be observed, such as the case of the fully connected representation, giving a lower efficacy of the reactive testing protocol compared to biweekly regular testing with 25% adherence, while the other representations yield the opposite ranking (see electronic supplementary material, S2.3.1). Figure 3*e*,*f* shows that the impact of a protocol on the distributions of epidemic sizes is also similar across representations: here, regular testing yields a strong reduction of the probability of having a large epidemic size and a higher peak at small sizes. We also show in electronic supplementary material, S2.3, how, when two protocols have similar efficacies, the resulting distributions of epidemic sizes are also very similar, and that this similarity holds across representations.

**Figure 4.**
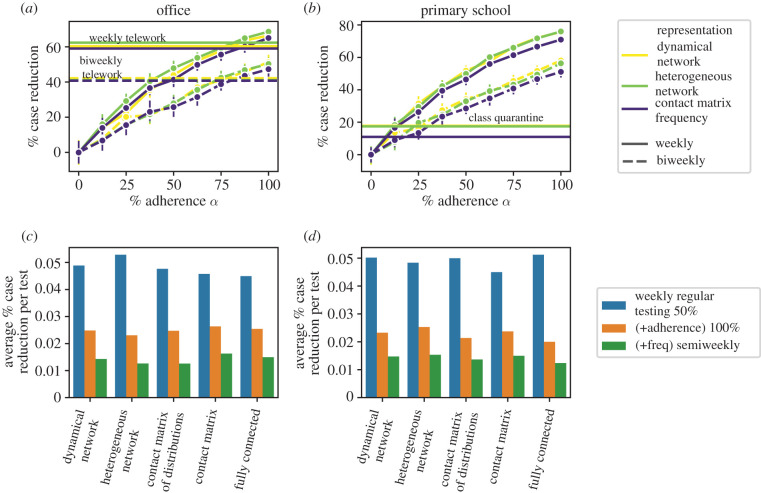
Effect of increasing adherence and frequency in regular testing protocols. (*a*) Effect of the adherence *α* for a given frequency (once per week or every two weeks) in the regular testing protocol for the office dataset and *R*_0_ = 1.5, compared with telework, for several data representations. Horizontal lines correspond to the performance of telework at the same frequencies. (*b*) Effect of the adherence *α* for a given frequency (once per week or every two weeks) in the regular testing protocol, compared with the class quarantine protocol, for the school dataset and *R*_0_ = 1.5. Horizontal lines correspond to the class quarantine protocol. (*c*,*d*) Effect of improving adherence or frequency, for *R*_0_ = 1.5 for office (*c*) and primary school (*d*). We consider weekly regular testing and α=50%, and we measure the average size reduction (with respect to symptomatic testing) per test (in blue), and the additional size reduction per additional test when doubling the adherence (in orange), and when doubling the frequency (in green).

We illustrate these results further in figure 4, where we investigate the question of the adherence to regular testing needed in offices to obtain the same efficacy as telework, for a given testing frequency (figure 4*a*). Although the value of the median size reduction obtained by telework slightly depends on the data representation (1 day per week of telework yields a 59±3% and 60±3% reduction for contact matrix and dynamical network representations, respectively), we estimate that regular testing with the same frequency becomes as efficient as telework for adherence values that remain similar across data representations, ranging from 84% (contact matrix representation) to 81% (dynamical network representation). Figure 4*b* considers instead the comparison between the regular testing and the class quarantine protocol: the estimation of the adherence needed for regular testing to become more efficient than class quarantine is also consistent across data representations. Another interesting point concerns the effect of increasing the number of tests, either by increasing adherence or by increasing frequency, within the regular testing protocol. First, the increase in efficacy faces diminishing returns (the efficacy grows less fast than proportionally to the number of tests). Second, and as already noted in [20] with simulations on the dynamical network representation of a school dataset, increasing adherence has a bigger impact than an increase in frequency (at equal additional number of tests). Figure 4*c*,*d* illustrates these points by showing the average size reduction per test for the weekly testing protocol with adherence 50%, and comparing it with the additional size reduction per test obtained for twice the number of tests, obtained either by doubling the adherence at the same frequency, or by doubling the frequency at the same adherence. We show in electronic supplementary material, S2.3.3, that this property holds in all settings, and for all data representations.

In electronic supplementary material, S2.3.2, we examine the impact of the reproductive number *R*_0_. As also observed in [20,48], the efficacy of each protocol depends in a non-monotonic way on *R*_0_. At small *R*_0_, even the symptomatic testing protocol leads to small epidemic sizes, so that additional protocols have a limited impact. At very large *R*_0_ instead, even the best protocols reach their limits and the spread cannot be well mitigated. These arguments hold for any data representation, and we indeed observe this non-monotonicity for all data representations. However, the optimal range of *R*_0_ depends on the data representation, with a larger value of the optimal *R*_0_ for the category-based representations. Moreover, the differences between the efficacy values of a given protocol by using different data representations become larger at large *R*_0_, with a larger estimated efficacy when using category-based representations.

Different protocols have different efficacies but also different costs, which need to be taken into account in decision-making processes. We thus compare in figure 3*c*,*d* the cost of each protocol simulated on each data representation, computed as the average number of days spent in quarantine per node. As for the efficacy, the precise evaluation of the cost depends on the data representation, but the ranking of protocols according to their cost does not (this is also true for the cost in terms of number of tests, as shown in electronic supplementary material, S2.3). In particular, regular testing at school avoids a large fraction of the number of days of class lost, with respect to reactive class closures. In the office, regular testing is more costly than telework, as the latter simply decreases the number of contacts without quarantining individuals.

Overall, figure 3 indicates that a coherent picture of the relative efficacy and cost of different protocols is obtained when using different representations of the data in the numerical simulations, even if quantitative differences in the precise evaluation are observed. Additional results shown in electronic supplementary material, S2.5, indicate that these conclusions are robust with respect to changes in disease and protocol parameters: even if the values of the efficacy and costs of each strategy depend on the parameters, and the ranking of strategies can even vary (e.g. for different values of the infectious period), this ranking remains independent of the data representation. We also explore in electronic supplementary material, S2.4, the combined effect of NPIs and vaccination. Using any data representation, vaccination alone reduces the final epidemic size even in the absence of NPIs or for the symptomatic testing protocol, and decreases the costs in terms of quarantines. Considering vaccination coupled to NPIs, results confirm the robustness of the ranking of protocols, when evaluated in terms of costs and benefits, highlighting the supplementary control that these strategies may have at intermediate vaccination coverages [20,44].

## 4. Discussion

We used high-resolution contact datasets to build aggregated representations and evaluate how the loss of resolution informing epidemic models can influence the evaluation of prevention and control strategies. Numerical simulations of a model for the spread of SARS-CoV-2 in educational and professional contexts show that detailed representations are needed to correctly account for over-dispersion of reproduction numbers and for an accurate evaluation of the efficacy and costs of each strategy. However, coarse representations containing only very summarized information are good enough to rank protocols, and thus to provide insights on better options given the context.

Models offer a unique opportunity to evaluate strategies for prevention and control of epidemics, anticipating their expected advantage and costs associated in order to inform public health decisions. Depending on the context and the question to be addressed, models need to integrate an accurate description of the population under study and of the contacts along which disease transmission occurs. In recent years, the increasing availability of datasets describing contacts between individuals has made it possible to devise models exposing the complexity of human interactions in terms of number of contacts, repeated contacts, frequency, duration, etc. For instance, models integrating data describing interactions with high temporal and spatial resolutions can be used to design and study measures tailored to specific contexts such as schools, where repetition of contacts because of friendships and structural organization of contacts due to classes impact the resulting epidemic dynamics [14,20,21,47]. Complex models are, however, data hungry, might be difficult to interpret, and are more time-consuming in terms of development and simulations. Moreover, detailed data are not always available, and datasets in specific settings may provide a narrow vision of the interaction patterns occurring in those contexts that may be difficult to generalize. By losing some of these specificities, aggregated representations may become more generally applicable.

Our results show that some differences emerge in the disease spread simulated on different data representations, even when calibrating the simulations to yield the same basic reproductive numbers. In particular, category-based representations tend to find a lower over-dispersion of the distribution of the reproductive number, and could thus lead to difficulties in correctly estimating the role of superspreading events. This is in line with recent results highlighting the role of contact heterogeneities in superspreading [37]. As they ignore individual differences, these representations cannot inform strategies targeted towards specific individuals, they are also less able to describe the cascading of a spread from individuals with a high connectivity to less well connected ones [51], and differ in the estimation of the final epidemic size [38].

The picture is more complex when dealing with the evaluation of control protocols. On the one hand, the ranking of protocols according to their efficacy or their cost does not depend on the data representation. The picture of which protocol is most efficient in each context remains coherent. When a protocol depends on several parameters, the information on which parameter is the most important to act upon is also coherent across data representations (e.g. increasing adherence for regular testing protocols has a larger impact than increasing frequency, at given number of tests). It is even possible to use coarse data representations to quantify the adherence needed for the regular testing to become more efficient than, for example, telework or class quarantine. On the other hand, using various data representations can lead to quantitative differences in the precise values of benefit and cost. This can be a limitation for coarse representations when decisions require accuracy in the estimate of the benefit/cost—for example, to define a minimum benefit that would trigger the application of the measure. Such decisions should thus take into account an inherent uncertainty in the model outcomes due to the limited information contained in the data.

We found that regular testing with high enough adherence is a very efficient strategy allowing to limit spread in school contexts while minimizing the number of lost school-days, confirming prior works [20,21,52]. In offices, telework is also very efficient [19]. Reactive class closure or reactive testing instead have limited efficacy. The robustness of such results across data representations is explained by the fact that these NPIs reduce the epidemic size through mechanisms that do not depend on the data description. Indeed, the efficacy of reactive measures is limited by the infectiousness of pre-symptomatic and asymptomatic individuals: for instance, due to the resulting silent propagation, many other classes can already have been reached by the infection when one class is closed upon the detection of a case at school [20]. By contrast, regular testing is a proactive approach that allows one to detect also pre-symptomatic and asymptomatic cases. Telework on the other side simply reduces the time in contact, reducing the probability of contagion events whatever the data representation. Overall, our results support the use of even coarse representations of the interactions between individuals in settings such as schools or workplaces when evaluating NPIs and potentially choosing between possible protocols.

Individual data such as the ones used in this study across different settings are rarely available. Moreover, the existing datasets are each specific to a context and potentially to the time of the data collection campaign. In emergency situations or during a crisis such as the current pandemic, gathering such data in real time encounters many challenges, and more coarse-grained representations are generally opted for. Indeed, summarized data are more accessible, and can be enriched by some robust statistical features of contact data, such as the heterogeneities in contact durations [30,35,38,43]. In particular, the division of a population into categories with, for example, different mixing patterns and/or schedules can be performed from limited information such as the existence of classes in a school or of departments in offices. A population can also be separated in groups according to an expected diversity of behaviours, as for instance in [44] that singles out the group of ‘more social’ students in a US campus as the ones belonging to fraternities and shows that targeted testing of this category can be an efficient strategy.

Our work comes with several limitations. First, the data we used describe contacts collected during only few days. Here, we have used the simplest method of repeating the dataset in order to simulate the contacts in the population during an extended time, whereas contacts are not repeated identically in the real world. However, the simulations performed in [20] used different ways of artificially extending the data duration and found no differences in the results. The settings we have considered are also relatively small, but represent the state of the art in terms of data describing interactions between individuals, and have very different structural and temporal properties because of structure and activities performed. More work needs to be done to generate synthetic datasets at such resolution in larger settings. Second, we used a rather simple coupling with the community, through regular introduction of cases, as the data we considered do not include contacts occurring outside of the studied context. This implies that we do not evaluate the impact of the interventions on the community: different approaches would be needed for this purpose [22,53], which, however, would lose resolution within each setting. Without going to such large-scale agent-based models, a possible improvement would be to inform the model with empirical data on the contacts that individuals have with members of the community, or with one another outside of school. Third, we have here considered one specific infectious disease. However, our results are robust with respect to variations in the basic reproductive number, initial immunity and the impact of vaccination. We have also explored a wide range of possible infectious periods, finding that it can affect the efficacy of measures and even their ranking, but that the ranking remains independent of data representation, at fixed infectious period (as already noted in [6,38], the precise order of contacts could affect the results for very fast processes whose timescales are of the same order as the temporal resolution). Moreover, SARS-CoV-2 is of particular interest both practically and theoretically, as the pre-symptomatic and asymptomatic transmissions make it necessary to go beyond the usual reactive strategies and to evaluate a range of protocols.

Our modelling approaches are agent-based, as the simulations consider distinguishable agents even when the data representations are category-based, which suggests two lines of further research. On the one hand, it would be interesting to extend our results to compartmental models. Indeed, the epidemic curves obtained in a free-spreading scenario by agent-based models and compartmental models can be mapped onto one another upon appropriate recalibration of parameters [40]. However, whether this remains the case when interventions are in place is an open question. On the other hand, the agent-based models we considered deal with the interactions between individuals but do not address the issue of individual heterogeneities with respect to the disease transmission (beyond the differences between children, adolescents, adults), such as heterogeneous infectious periods [54] or heterogeneous rates of transmission [55], nor with respect to potential changes of behaviour depending on the epidemic situation [56]. An interesting extension of this work would be to consider situations where these differences between individuals are correlated with their contact behaviour: to take into account such correlations, one would need to go beyond the category-based representations we have considered here, allowing heterogeneous properties within each category, in the spirit of degree-corrected stochastic block models [57].

## Data Availability

We use publicly available datasets. The original datasets can be accessed at http://www.sociopatterns.org/datasets and the datasets and the code used in the paper are available at https://github.com/diegocontr/EpidemicSimulation [58]. Electronic supplementary material is available online [59].
